# Obsessive compulsive disorder in treatment seeking children & adolescents during the COVID-19 pandemic

**DOI:** 10.1192/bjo.2021.134

**Published:** 2021-06-18

**Authors:** Anthony Henein, Ana Pascual-Sanchez, Suzana Corciova, Matthew Hodes

**Affiliations:** 1 Imperial College School of Medicine; 2 Centre for Psychiatry, Imperial College; 3 Royal Free Hospital; 4 Centre for Psychiatry, Imperial College, Westminster CAMHS, Central and North West London NHS Foundation Trust

## Abstract

**Aims:**

Few studies have investigated the COVID-19 pandemic's effect on children and adolescents with obsessive compulsive disorder (OCD), who are thought to be particularly vulnerable. This study aims to investigate whether the COVID-19 pandemic is associated with increased referral of young people with OCD in one area of London and determine if COVID-19 has been associated with change in symptom severity and treatment offered in recent years.

**Method:**

A retrospective study was conducted using clinical service data investigating 58 young people (8–17 years) referred and assessed in CNWL NHS Foundation Trust CAMHS, before and during the COVID-19 pandemic in 2020 (months March–October 2018–2020). Changes in symptom severity were measured using the health of the nation outcome scale for children and adolescents (HoNOSCA). Total HoNOSCA and three HoNOSCA items were used; emotional symptoms, family relationships and school attendance. Patient clinical records were reviewed to assess if COVID-19 had exacerbated OCD symptoms. The type of treatment offered (cognitive behavioural therapy -CBT- only vs combined CBT and medication) was also compared. Analysis was carried out using Chi-square, Kruskal–Wallis and Mann–Whitney U tests.

**Result:**

26 (5.62%) initial assessments to CAMHS were related to OCD in 2020, compared to 12 (1.30%) and 20 (2.27%) assessments pre-pandemic (2018 and 2019), showing a significant increase in the proportion of OCD cases (X2 (1, N = 58) = 20.3, p < .001). There was no significant difference in total HoNOSCA, emotional, family relationships, or school attendance scores on initial assessment. However, 69.2% of clinical records in 2020 showed symptom worsening over the COVID-period, compared to 30.8% of cases assessed pre-pandemic.There was a significant difference between the type of treatment offered before and during COVID-19 (X2 (2, N = 58) = 12.7, p = .002), with a higher proportion of patients who were referred to CAMHS for OCD but discharged without treatment before the pandemic (37.5% vs 0%). While CBT only remains the most frequent treatment offered, combined treatment was more frequent during the pandemic, although this difference was not significant.

**Conclusion:**

The proportion of OCD-related initial assessments in CAMHS increased during the pandemic despite the overall number of referrals falling. Furthermore, fewer cases were discharged without treatment in CAMHS during this period. Given this, and that many were reported to have deteriorated during the pandemic, services will likely need to address the increased burden of more severe cases. Further research is warranted to assess the generalisability of our findings.

